# Compressive and Tensile Elastic Properties of Concrete: Empirical Factors in Span Reinforced Structures Design

**DOI:** 10.3390/ma14247578

**Published:** 2021-12-09

**Authors:** Alexander Sergeevich Korolev, Anastasia Kopp, Denis Odnoburcev, Vladislav Loskov, Pavel Shimanovsky, Yulia Koroleva, Nikolai Ivanovich Vatin

**Affiliations:** 1 Department of Building Construction and Structures, South Ural State University, 454080 Chelyabinsk, Russia; anastasiya.kopp@mail.ru (A.K.); livedisa154@gmail.com (D.O.); wlad_loskow@mail.ru (V.L.); Shiman_97@bk.ru (P.S.); korol_14@mail.ru (Y.K.); 2 Self-Healing Structural Materials Laboratory, Peter the Great St. Petersburg Polytechnic University, 195251 St. Petersburg, Russia

**Keywords:** multi-physics models, modulus of elasticity, concrete, deformability, compressive modulus of elasticity, tensile modulus of elasticity, non-linear calculation, span structures

## Abstract

Concretes with the same strength can have various deformability that influences span structures deflection. In addition, a significant factor is the non-linear deformation of concrete dependence on the load. The main deformability parameter of concrete is the instantaneous modulus of elasticity. This research aims to evaluate the relation of concrete compressive and tensile elastic properties testing. The beam samples at 80 × 140 × 1400 cm with one rod Ø8 composite or Ø10 steel reinforcement were experimentally tested. It was shown that instantaneous elastic deformations under compression are much lower than tensile. Prolonged elastic deformations under compression are close to tensile. It results in compressive elasticity modulus exceeding the tensile. The relation between these moduli is proposed. The relation provides operative elasticity modulus testing by the bending tensile method. The elasticity modulus’s evaluation for the reinforced span structures could be based only on the bending testing results. A 10% elasticity modulus increase, which seems not significant, increases at 30–40% the stress of the reinforced span structures under load and 30% increases the cracking point stress.

## 1. Introduction

Concrete is a complex multi-scale composite involving multi-physics processes of its hardening and deformations. Elastic modulus is an important mechanical parameter for measuring the stiffness of concrete members and structural design [1,2]. The base principle in the design of reinforced concrete structures by deformations is equality of concrete compressive and tensile elastic properties. It concludes in [3,4]:

1. the viscoelastic character of concrete deforming under load;
2. equal deforming under equal stresses;
3. three-line model of non-linear deforming under load.

The main concrete elasticity parameter is elasticity modulus or Young’s modulus. It can be obtained by the following methods [1,5,6]:

4. axial compression deformation test;
5. calculated by the multi-scale model and homogeneity theory based on the micro-elastic properties by the nanoindentation test;
6. evaluated by the relationship between the compressive strength or the dynamic elastic modulus and the static elastic modulus;
7. use of ultrasonic waves.

The standard compression test considers only instant elastic deformations, so this elasticity modulus is instantaneous [7,8]. Many factors of concrete elasticity modulus formation were researched. The elasticity modulus of concrete depends on aggregates’ maximum size [9,10].

In [11], the effect of the interfacial transition zone, where the cement paste meets the aggregate surface, on the elasticity modulus of cement concrete was investigated. The effect of this zone’s thickness and elastic modulus on the elastic properties of cement concrete is shown to be significant. It is found that a larger thickness of interfacial transition zone with a relatively low elastic modulus has a more noticeable effect on the elastic modulus of concrete, and the effect of interfacial transition zone thickness is negligible when the elastic modulus of the interfacial transition zone is large enough. The influence of the interfacial transition zone’s local micro-properties on the behaviour concretes was studied for concretes made with recycled ceramic coarse aggregate additives [12]. The results show that the minimum and mean values of the elastic modulus and the interfacial transition zone thickness impact concrete mechanical and elastic properties to different degrees.

The three-phase model is proposed for the random concrete microstructure using the Voronoï tessellation to evaluate the interfacial transition zone volume fraction in concrete analytically [13]. The interfacial transition zone volume fraction was found not to exceed 7% for typical concretes. It is found that the concrete elastic modulus increases with increasing aggregates volume fraction, aggregates maximum size and the proportion of coarse aggregates and with decreasing the interfacial transition zone thickness and elastic modulus.

The review [14] summarised the interfacial transition zone approach. The dependence of interfacial transition zone thickness on aggregate’s shape and the influence of the sphericity of particles on the elastic modulus was presented. All the reviewed investigations show that particle shape significantly affects the microstructure and properties of cementitious composites [15].

A homogenisation scheme of concrete was developed to estimate the effective elastic moduli of a hydrating cement paste [16]. The homogenisation theory for disordered media was used to estimate the evolution of the effective elastic moduli of the hydrating paste. The model thrived predicts the evolution of elastic modulus of a cement paste at a late age. Sanahuja et al. conclude that the task that remains to be carried out consists of dealing with nonlinear phenomena necessary for addressing strength.

Static and dynamic modulus of elasticity significantly depends on the component composition of the concrete. An example is research [17] that presents the results of static and dynamic modulus of elasticity measurements on high-performance concretes with partial replacement of cement by metakaolin, microsilica and fly ash. The destructive compression test determined static modulus. The highest values were reached for fly ash at 20% and 30% replacements. An agreement between dynamic and static modulus dependence on cement replacement level was observed.

In [18], different models were compared to predict the elastic properties of slag concrete. The Reuss model was evaluated as a precise model for predicting low strength slag concretes containing low cement content, high w/c ratio or high slag replacement ratio. Voigt, Hashin–Hansen and Hirch-Dougill models can also be used for estimating the modulus of elasticity of high strength concrete. Voigt model obtains the closest estimations for high strength slag concretes or low permeable.

The mechanical properties of concrete are highly dependent on the properties and proportions of binders and aggregates. More than 3000 components of experimental data on the relationship between concrete compressive strengths and modulus of elasticity were collected in [19] and analysed statistically. As a result, a practical and universal equation was proposed, which considers types of coarse aggregates and types of mineral admixtures.

The [20] reviews the influence of incorporating recycled aggregates, sourced from processed construction and demolition waste, on the modulus of elasticity of concrete. The 588 concrete mixes were statistically analysed. The loss of modulus of elasticity was statistically analysed based on recycled aggregates’ quality and replacement level. The modulus of elasticity normally decreased with increasing recycled aggregates content. The degree of modulus of elasticity depends on the original material’s type, size, and quality. For a given compressive strength, most studies obtained moduli of elasticity of recycled aggregates concrete exceed the curve for sandstone aggregates proposed in Eurocode-2. This exceedance means that even when high replacement levels to recycled aggregates are used, the resulting recycled aggregates concrete would generally have moduli of elasticity compliant with existing standards and specifications for natural aggregate concrete.

Static and dynamic methods have tested the elasticity modulus of concrete samples of different compositions [21]. The investigation of self-compacting concrete mixes demonstrated that concrete with natural aggregates needs significantly less water to achieve the required flowability of a concrete mix than concrete with crushed basalt aggregate. The elasticity modulus of concretes testing demonstrated that values obtained with dynamic testing are higher than those obtained with the static testing method. The results of concretes testing (self-compacting concrete and high-performance concrete) show that differences between static and dynamic elasticity modulus are lower with an increased volume content of coarse aggregate.

In [5], the influence of moisture content on the elasticity modulus was analysed. As a result of the development of micro-cracks in the transition region during drying, the elasticity modulus decreases with the moisture content increasing. The elasticity modulus is 30% higher for fully saturated concrete than dry concrete. If the moisture contents are almost similar, then the elasticity modulus of the specimens cured under the natural conditions reduces slightly because concrete develops incompletely in the curing stage, leading to the development of micro-cracks. Based on the experimental data and the analytical results, a formula indicating the relationship between moisture content and the elasticity modulus of concrete was proposed.

The use of local materials for developing ultra-high performance concrete (UHPC) possibly decreases the elasticity modulus of ultra-high performance concrete. In [22], the equation was proposed for predicting the ultra-high performance concrete mixtures containing local materials. The inclusion of industrial waste in concrete significantly affects the elasticity modulus. The dynamic modulus of elasticity and durability of concrete could be improved by adding waste glass powder [23]. In contrast, incorporating rubber particles in concrete mix can degrade mechanical (modulus of elasticity, compressive strength, tensile strength and flexural strength) properties [24]. An even more complex determination of the modulus of elasticity of composite materials, for example, concrete-filled tubes [25].

However, in practice, there is no material operatively in-controlled or out-controlled by elasticity parameters. At first, it is related to the testing method’s hardness and long-term sensor and centre installation. Due to that, the three-point bending method of elasticity testing by the only deflection definition appeared simple, available, and accurate. There are few recent studies [1,26] on the modulus of elasticity in tensile elasticity modulus since this problem is considered solved and not relevant. However, empirical results and works in the theory of non-isotropic elasticity show the opposite. The results showed an underestimation of the elastic modulus for conventional concrete in the majority of the existing models [27].

The continuous damage mechanics theory proposes an elastic bimodulus creep damage constitutive model is proposed in [28] based on the continuous damage mechanics theory. Ambartsumyan bimodulus theory was used [29]. The proposed model suppose to describe the damage-induced unilateral behaviour related to the microdefect closure effect. Numerical calculations validated the model by comparing the results with the traditional model’s results. It is demonstrated that the proposed model could describe the damage-induced unilateral behaviour related to microcracked closure effects. However, a comparison of the model with experimental data on concrete deformation was not made.

The main imperative of the isotropic theory of elasticity about equality of concretes’ compressive and tensile elasticity demands modern retesting. The observed differences between concrete’ compressive and tensile deformative properties and design and control consequences determine the novelty of this study.

Thus, this research aims to evaluate the relation of concrete compressive and tensile elastic properties testing, taking into account non-linear deformability and effectiveness of elasticity modulus increasing in reducing bending concrete elements deflections.

Research tasks were concluded in empirical research of concrete elastic properties and its data testing in design:

1. Research the concrete deformability on standard beam samples with the equal strength of concrete and different elastic properties under compression and bending tensile;
2. Research the deformations of concrete beams, reinforced by steel and composite, under bending load;
3. Modelling and deflection calculation of the same beams under the same load conditions;
4. Estimation of elasticity modulus increasing effectiveness in bending reinforced concrete elements exploitation.

## 2. Materials and Methods

**Figure 1:**
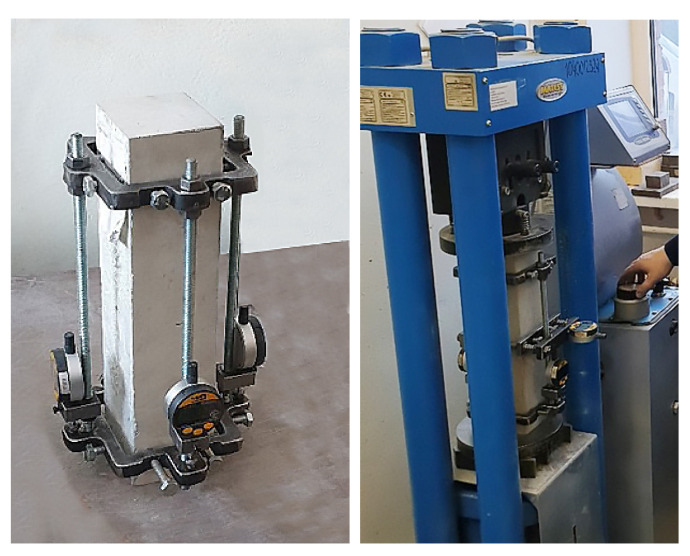
Concrete sample modulus of elasticity under compression testing.

Standard methods of Russian State Standards GOST 24452-80 “Concretes. Methods of prismatic, compressive strength, modulus of elasticity and Poisson’s ratio determination” [30] and Russian State Standards GOST 22690-2015 “Concretes. Determination of strength by mechanical methods of nondestructive testing” [31] were used to determine the modulus of elasticity and compressive strength of concrete of various classes. Each classes series of 3 samples was tested under compression on Matest press to determine longitudinal elastic and plastic deformation using digital deformation sensors on every side of the sample. Compression was made by 10% cracking stress stages to 40% cracking stress (Figure 1). Elasticity modulus has been determined as a relation of 30% cracking stress to the sum of elastic relative deformation except for plastic on stages delay by the standard. Compressive strength was determined on 6 samples series by the standard.

**Figure 2:**
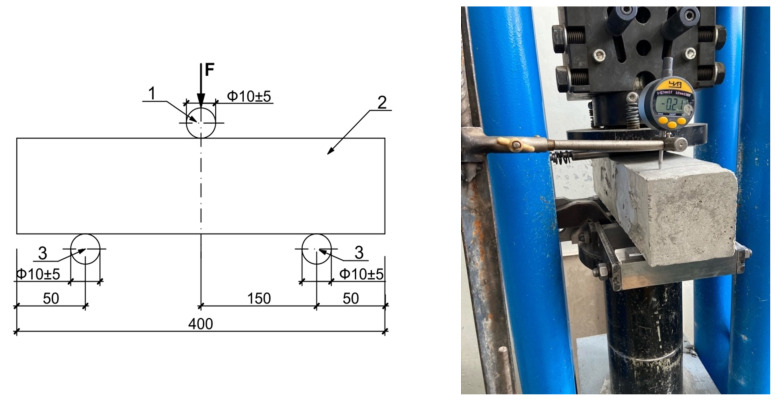
Bending tensile testing scheme 1—loading element; 2—beam sample; 3—support.

There is no standard testing method of bending tensile elasticity modulus of concrete. This testing was carried out on the base of small cement beams (5 × 5 × 15 cm) by Russian State Standard GOST 310.4-81* “Cements. Мethods of bending and compression strength determination” [32]. Mechanical loading was made by stages in 1.5 kN with 5 min carrying until breaking. The sample has been installed on the support faces of special utility in horizontal orientation under the testing machine. Figure 2 shows the testing scheme and photo. The loading rate was 50 ± 10 N/s.

Bending tensile elasticity modulus ***E_bt_*** was determined by the famous based on Mor integral theory of elasticity’s equation [29,33]:

(1) $$E_{bt}=\frac{Fl^{3}}{4bh^{3}f}$$

where ***F*** is the load, ***l*** is the two-point size, ***b*** is the sample width, ***h*** is the sample height, ***f*** is the deflection.

The elasticity modulus of cement hydrated paste was determined on the polymer plate samples testing device. Cement hydrated paste samples 70 × 5 × 5 mm were used.

**Figure 3:**
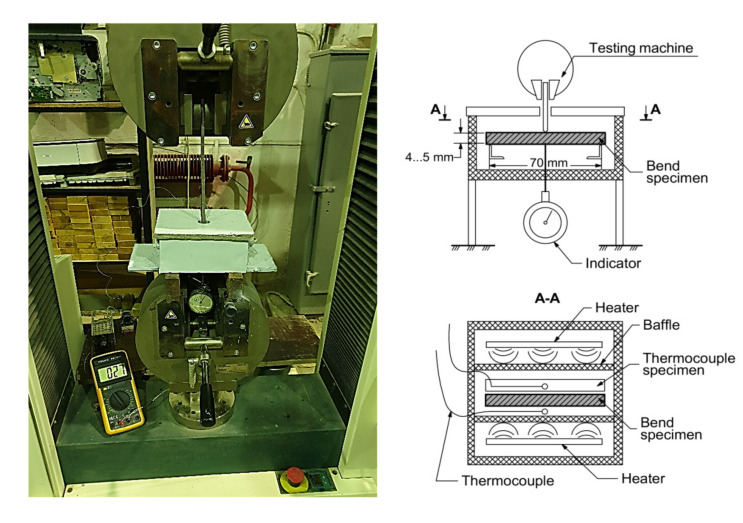
The three-point bending test rig.

According to the producer’s data for the Tinius Olsen h100ku machine, the load accuracy was ± 0.5% in the range from 0.2–100% of the installed load sensor (100 kN). The resolution of measuring the crosshead’s movement is 0.1 mm with an error of up to 0.01 mm. The centre point displacement of the subjected load’s sample was monitored by a mechanical dial gauge mounted on the small test chamber’s bottom. This monitoring was aimed to exclude the machine compliance influence. The difference between the displacements’ readings along the traverse and the dial gauge did not exceed 2% (Figure 3).

**Figure 4:**
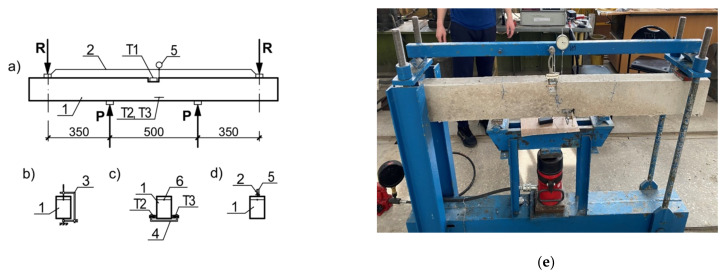
Beam testing scheme. (**a**) load and device placing; (**b**) strain gauges T1 fixing; (**c**) strain gauges T2, T3 fixing; (**d**) deflection indicator fixing; 1—beam; 2—deflection measurement traverse; 3, 4—strain gauges T2, T3 fixing; 5, 6—deflection indicator; (**e**) beam testing photo.

In research of concrete elasticity increasing effectiveness, the beam samples 80 × 140 × 1400 cm with one rod Ø8 composite or Ø10 steel reinforcement were made. The beam testing scheme and photo are shown in Figure 4. Strain gauge T1 is for reinforcement rod deflection measurement, I1 is an indicator of deflection, strain gauges T2 and T3 are for compression deformation of concrete measurement.

All bending tensile tests were made using each classes series of 3 samples.

**Figure 5:**
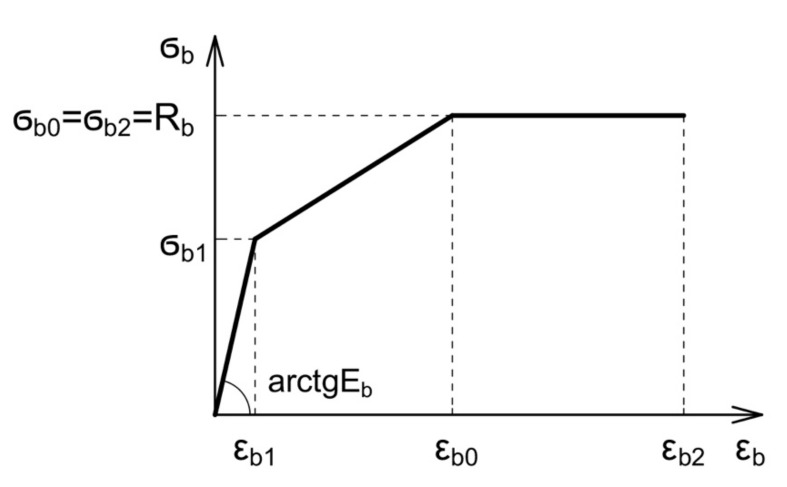
Three-line concrete deformation diagram.

Reinforced concrete beams were modelled in the FEM software LIRA-SAPR. This software provides linear and non-linear calculations. Non-linear schemes were used for calculations of concrete structures deflection. The non-linear three-line concrete deformation code is used (Figure 5).

The industrial concrete mixes with regulated elasticity modulus for samples were made on automatic mixing plant PK SCM Ltd. (Russia, Chelyabinsk) from local producers materials. Granite coarse aggregate 5–20, 10–20 mm fractions, quartz sand and Portland cement B42.5 CEM I were used for heavyweight concrete (HWC). Expanded clay gravel, quartz sand, perlite sand, and Portland cement B42.5 CEM I for lightweight concrete (LWC). Materials proportions were received based on authors’ previous works [33]:

1. the HWC B25 with normal elasticity modulus E = 30,000 MPa (concrete 1);
2. the HWC B25 with increased elasticity modulus E = 33,000 MPa due to fractioned coarse aggregate 10–20 mm (concrete 2);
3. the LWC B15 D1600 with E = 15,000 MPa on the base of expanded clay gravel and quartz sand (concrete 3);
4. the LWC B7.5 D1000 with E = 5000 MPa on the base of expanded clay gravel and perlite sand (concrete 4).

The hydrated cement paste samples were made from cement paste of normal density.

## 3. Results and Discussion

### 3.1. Research of the Standard Sample Concrete Beam Deformability with Different Elastic Properties under Compression and Tensile Bending

**Figure 6:**
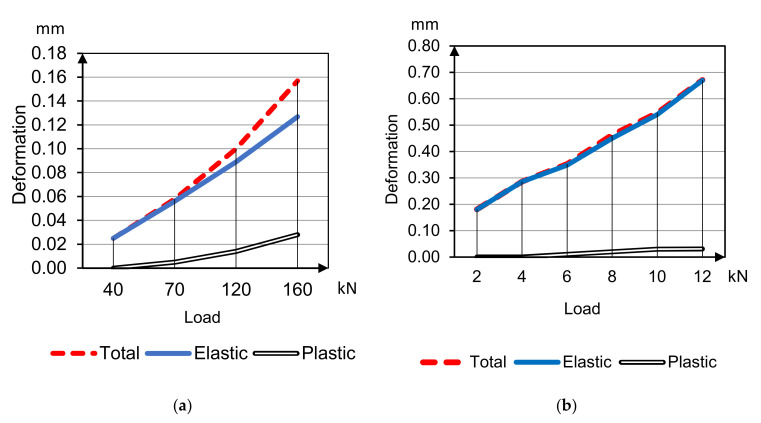
Deformation and deflection curves for concrete 1 under compression (**a**) and bending (**b**) tensile.

**Figure 7:**
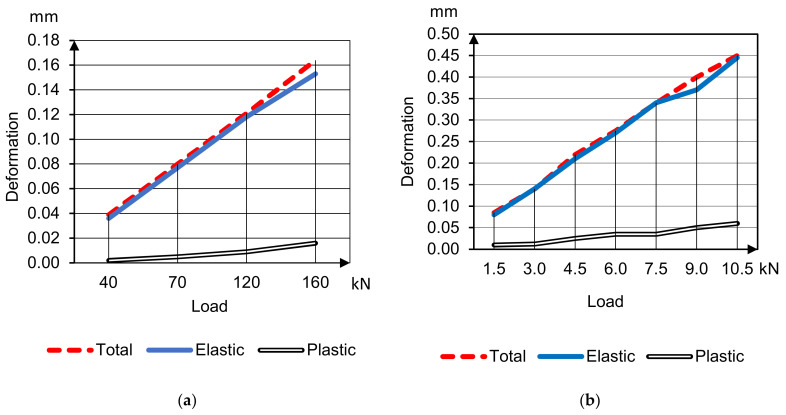
Deformation and deflection curves for concrete 2 under compression (**a**) and bending (**b**) tensile.

**Figure 8:**
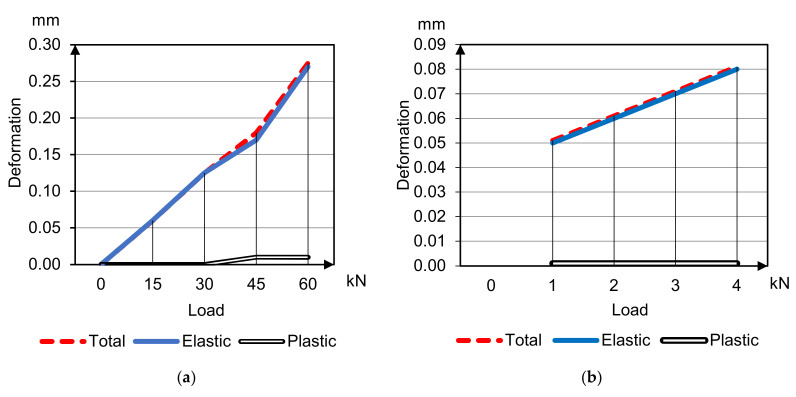
Deformation and deflection curves for concrete 3 under compression (**a**) and bending (**b**) tensile.

**Figure 9:**
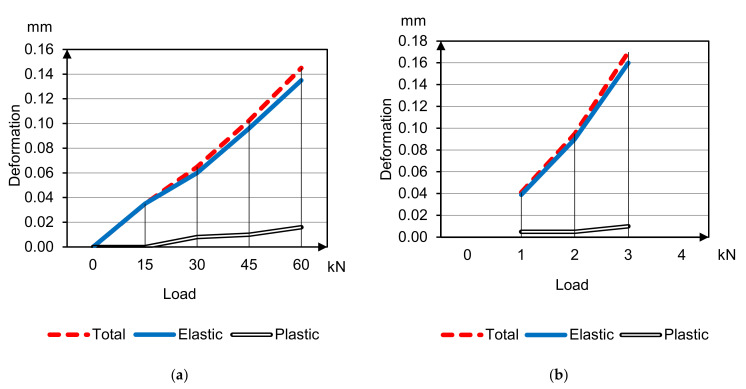
Deformation and deflection curves for concrete 4 under compression (**a**) and bending (**b**) tensile.

The average deformability parameters of sample beams 400 × 100 × 100 mm under compression and bending are presented in Figure 6 and Figure 7 of HWC (concretes 1, 2 accordingly); and Figure 8 and Figure 9 of LWC (concretes 3, 4 accordingly). The plastic deformations are short-termed and determined by results after 5 min delay on each loading stage. In these tests the loading was going to 30–40% strength without destruction for only deformative properties determination.

Curves’ analysis clears specifies of different concrete types deformation under compression and bending tensile

1. Plastic deformations under compression on the load stages delay begin to develop already after reaching 20% of breaking load, and with the load increasing plastic deformations’ part grows, and total modulus of deformation degrades in arithmetical progression keeping instantaneous elasticity modulus;
2. Plastic deformations under tensile bending are minimal and do not develop until concrete destruction;
3. Plastic deformations of concrete on porous aggregates are much less compared to heavyweight concretes.

These facts prove the difference between deformation and destruction mechanisms under compression and tensile. In addition, it points to the solving significance of hydrated cement paste and aggregates contact zone (ITZ) in deformation under compression process [11,12,13].

**Figure 10:**
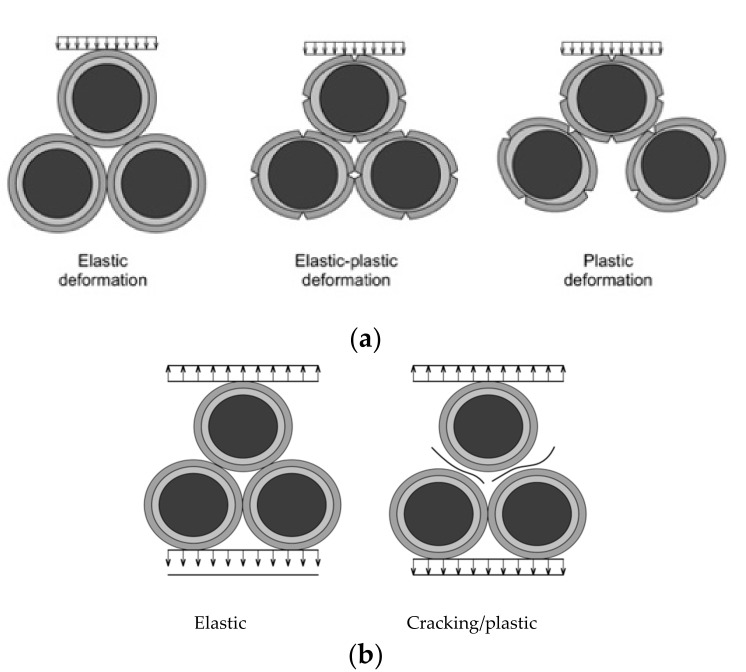
Stages of concrete deformations until destruction. (**a**) under compression three stages, (**b**) under tensile two stages.

Deformation’s process under compression developing up to 20% breaking load is the process of developing micro-cracking with stress relaxation by the contact zone and, as a result, delayed macro-cracking until total destruction. The micro-cracking and macro-cracking process under tensile is developing very quickly right before the destruction until that structure resists elastically. These ideas are presented in destruction under compression and tensile models in Figure 10.

Table 1 shows the elasticity and deformation modulus of concretes and hydrated cement paste (HCP) under compression and bending tensile.

The test results showed that bending tensile elasticity modulus is several times less than compression elasticity modulus for concrete as the hydrated cement paste. Thus, the difference between compressive and tensile deformations concludes in hydrated cement paste properties [24,25].

### 3.2. Research of Concrete Sample Beams Reinforced by Steel and Composite under Bending Tensile

**Figure 11:**
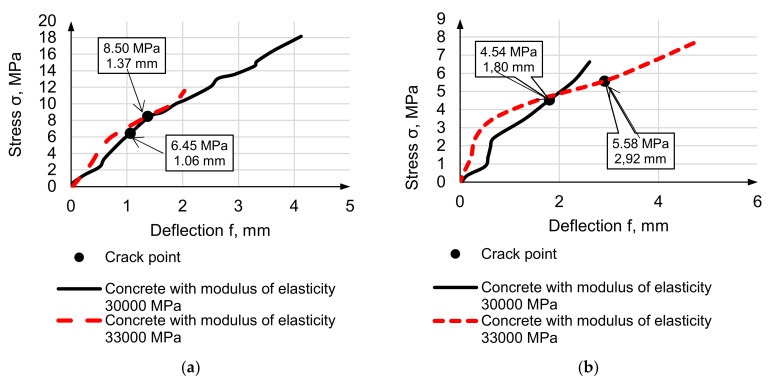
Stress-deflection curves for HWC, reinforced by steel (**a**) and composite (**b**).

**Figure 12:**
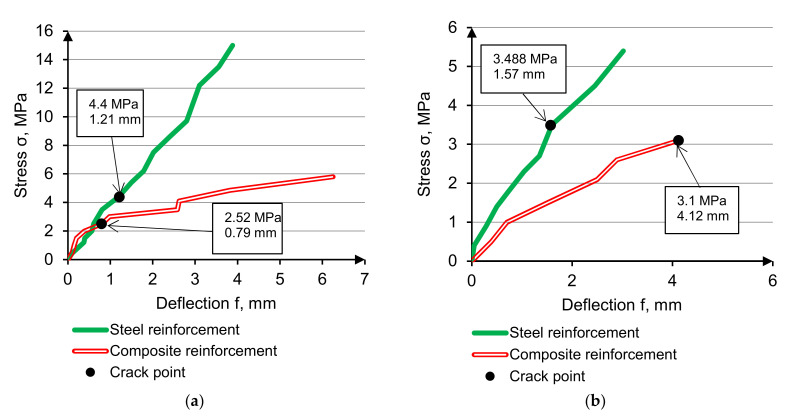
Stress-deflection curves for LWC D1600 concrete 3 (**a**), D1000 concrete 4 (**b**), reinforced by steel and composite.

Figure 11 and Figure 12 present the stress curves depending on deflection under bending load. During the test, the samples were loaded until cracks appeared and continued after cracking until beams’ full destruction.

The important feature is that increasing the elasticity modulus of equal-strength concretes’ results in proportional and not significant deflection decreasing, but non-proportional cracking point stress increasing. 10% elasticity modulus growth accords to 30% cracking point stress growth. Finally, the cracking point stress of high-modulus concrete with low-modulus reinforcement is close to the cracking point stress of normal concrete with high-modulus steel reinforcement. Cracking point stress of lightweight concretes decreases accordingly to elasticity modulus decreasing but not to strength.

**Figure 13:**
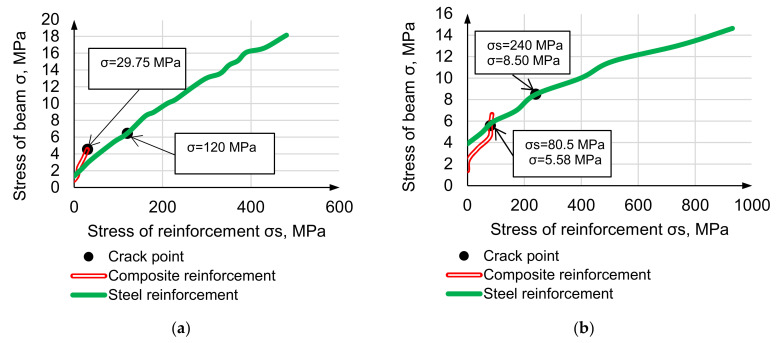
Beam stress-reinforcement stress curves of HWC, reinforced be steel (**a**) and composite (**b**).

Beam stress-reinforcement stress curves are presented in Figure 13.

Reinforcement stress demonstrates that elasticity modulus increasing results in reinforcement’s involvement level in total resistance growth. On cracking point, steel stress/normal concrete stress ratio is 18.6, steel stress/high-modulus concrete stress ratio is 28.2, composite stress/normal concrete stress ratio is 6.6, composite stress /high modulus concrete stress ratio is 14.4. Reinforcement involving resistance is 1.5…2 times higher in high-modulus concrete, and the effect grows using low-modulus reinforcement.

By design LIRA-SAPR software, the non-linear deflection calculation was made based on the three-line deformation diagram and equality of compressive and tensile deformations principal. The deflection was calculated with cracking point loads for each variant. Calculation results are presented in Table 2.

As it can be seen, calculated deflections are much more depressive than fact. It proves that the three-line deformation diagram in tensile results in deflections’ calculation mistakes. The two-line elastic scheme is much more accurate.

The empirical results of this research point to the concrete’s bimodularity properties. These results could be used to develop the elastic bimodulus creep damage constitutive model proposed in [28]. Classical elasticity theory assumes that materials have the same elastic properties in tension and compression. Even so, this is only a simplified interpretation and does not account for material nonlinearities [34,35]. The bimodular materials’ properties approach with moduli under tensile loading, which are different from those under compressive loading, is used for models in the form of stress-strain or constitutive relations [36].

Many investigations included bimodular materials’ properties modelling on the base, in first, the models of Ambartsumyan [29]. However, the concrete’s bimodularity is considered too rarely. In the case of our study, the main is the linearity of tensile deformations in a difference of non-linear compression deformations’ development. That is so important in effective span structures design.

Based on the elastic theory for different elastic modulus at tension and compression, the analytical solution was deduced for the bending-compression column subject to combined loads [37]. As a result of considering two different moduli, the tension stress diminish with the increase of compression modulus, while the compression stress increases. It is concluded that the formula of classical mechanics is not applicable for calculating structure with different materials’ elastic moduli. We can also adjust the stress of concrete structure using a material with different moduli and reduce the maximum adverse tension stress by increasing compression modulus.

The analytical and empirical results proved that all kinds of concretes under tensile deform elastically until destruction. Concrete deformation under compression has a classic visco-elastic character beginning to 20% of breaking stress. Plastic deformation’s part is much less in lightweight concretes on porous aggregates than heavyweight concretes. Thus, the main plasticity factor is the porous hydrated cement paste’s aggregate contact zone, which in lightweight concrete is much denser. Under tensile, the porous contact zone cannot be the reserve for plastic deformations, so the deformation character becomes definitely elastic.

Bending tensile tests showed that the tensile modulus of elasticity is several times lower than compressive; the same is for hydrated cement paste. It proves that hydrated cement paste structure under compression is more resistant than tensile because of inner friction between particles and layers.

The simple Equation (2) of compressive/bending tensile modulus of elasticity relation can be suggested

(2) $$E_{c}=k_{c}E_{tens}$$

where $k_{c}$ is the proportional coefficient for heavyweight and lightweight concretes. The coefficient $k_{c}\;$ equals 10 if the density exceeds 1200. For lightweight concretes, the coefficient $k_{c}\;$ equals 5 if the density is less than 1200.

It could be the base of the simple operative elasticity modulus test method of three-point bending tensile by the deflections under load.

It is empirically proved that increasing equal-strength concrete’ elasticity modulus significantly influences the cracking point stress of reinforced structure but not on the deflection. Modulus of elasticity 10% increasing results in 30% cracking point stress growth due to lower plastic deformation of concrete with higher elasticity in contact adhesive zone to reinforcement and higher involving level of reinforcements in concrete tensile resistance. In practice, these effects could be achieved only in the case of regulation, coding and operative control of industrial concrete elasticity modulus.

## 4. Conclusions

This study determined the difference between concrete’s compressive and bending tensile deformative properties so as consequences of this difference in elastic properties evaluation and considering in reinforced span structures design. Based on the results obtained, it is possible to draw the following conclusions.

1. Concrete tensile deformations’ character is elastic, not depending on the load level in contrast with the viscoelastic character of deformations under compression. The concrete deformations’ models under compression and tensile are proposed.
2. The three-line deformation model of span structures is ineffective. This model exceeds calculated deflections and over reinforcement in span structures design. The elastic two-line model is more applicable than the three-line deformation model.
3. Instantaneous elastic deformations under compression are much lower than tensile. Prolonged elastic deformations under compression are close to tensile. It results in compressive elasticity modulus exceeding the tensile. The relation between these moduli is proposed. The relation provides operative elasticity modulus testing by the bending tensile method.
4. The elasticity modulus’s evaluation for the reinforced span structures could be based only on the bending testing results. A 10% elasticity modulus increase, which seems not significant, increases at 30–40% the stress of the reinforced span structures under load and 30% increases the cracking point stress.

## Figures and Tables

**Table 1:** Deformative properties of concretes.

N°	Modulus of Elasticity under Compression E_c_, MPa	Modulus of Deformation under Compression E_cd_, MPa	Modulus of Elasticity under Bending Tensile E_bt,_ MPa	Modulus of Deformation under Bending Tensile E_btd,_ MPa
1	30,000	18,000	2900	2600
2	33,000	20,800	3375	3000
3	15,180	14,500	1550	1550
4	5800	5800	1100	1050
HCP	50,000	-	5860	-

**Table 2:** Calculated and fact deflections of concrete beams.

Concrete N°/Reinforcement	Bending Tensile Modulus of the Reinforced Beam, MPa	Calculated Deflection, mm	Fact Deflection, mm	Deflection Provision, %
1/steel	13,400	3.39	1.41	140
1/composite	7200	9.74	1.80	441
2/steel	14,500	3.34	1.37	144
2/composite	8500	9.59	1.57	510
3/steel	9100	3.09	0.67	361
3/composite	6200	4.30	0.79	544
4/steel	5900	4.75	1.35	251
4/composite	3300	12.1	4.12	294

## Data Availability

The data presented in this study are available on request from the corresponding author.
